# Editorial Note: Global Regulator SATB1 Recruits β-Catenin and Regulates T_H_2 Differentiation in Wnt-Dependent Manner

**DOI:** 10.1371/journal.pbio.3003507

**Published:** 2025-11-20

**Authors:** The PLOS Biology Editors

Following the publication of the Expression of Concern on this article [1,2], concerns were raised about regions of similarity within the first two lanes in the left panel of Fig S2A of [1], and that [1] states that “thymocyte viability was not significantly reduced even without using the OP9-DL1 co-culture system (Figure S5A)” without viability percentages or statistical test results in the text or figure legend.

As noted in [2], the images provided in S1 File in [2] to support the left western blot panel of Figure S2A in [1] do not appear to match the published figure panel and did not clarify the concerns about discontinuities or similarities in the published figure. The published figure has a stronger and clearer interaction signal than the underlying images; however, the underlying images – while different from the published figure – show a band in the position that would indicate an interaction, and support the conclusion.

The corresponding author responded to state the following:

- In Figure S5A in [1], the data represent the percentage viability of thymocytes co-cultured with OP9 or OP9-DL1 cells under control, Dkk1, and Wnt 3A treatment conditions.
- The statement “thymocyte viability was not significantly reduced even without using the OP9-DL1 co-culture system” refers to a separate cell death assay performed on thymocyte cultures (presented in Figure S5B in [1]).
- The MTT assay readings (Figure S5A in [1]) were obtained using a spectrophotometer, and cell death measurements (Figure S5B in [1]) were performed using a hemocytometer under a phase contrast microscope.
- All measurements in Figure S5 in [1] were manually recorded and the data underlying Figure S5 are no longer available.
- The viability percentages are presented on the y-axes of Figures S5A and B in [1] and statistical significance for these observations was assessed using one-way ANOVA, with a *p*-value threshold of 0.01 considered significant for viability assays.The Figure S5 assays in [1] were conducted to confirm that no substantial differences in cell viability existed between cultures under the experimental conditions used.

The *PLOS Biology* Editors issue this Editorial Note to provide an update to the Expression of Concern [2] previously issued on this article [1] and to provide readers with the additional information provided about Figure S5.
